# LC-MS/MS Method for Therapeutic Drug Monitoring of Abiraterone, Darolutamide, Apalutamide, Enzalutamide, and Metabolites in Prostate Cancer Patients

**DOI:** 10.3390/ijms27073017

**Published:** 2026-03-26

**Authors:** Bianca Posocco, Diletta Pasin, Nicoletta De Cesaro, Alice Pivetta, Sara Gagno, Giovanni Canil, Eleonora Cecchin, Riccardo Cecchin, Sara Speziani, Arianna Dri, Giorgia Bortolus, Michele Spina, Sandra Santarossa, Fabio Puglisi, Lucia Fratino, Erika Cecchin

**Affiliations:** 1 Experimental and Clinical Pharmacology Unit, Centro di Riferimento Oncologico di Aviano (CRO), IRCCS, 33081 Aviano, Italy; diletta.pasin@studenti.units.it (D.P.); nicoletta.decesaro@studenti.unipd.it (N.D.C.); alice.pivetta@studenti.unipd.it (A.P.); sgagno@cro.it (S.G.); giovanni.canil@cro.it (G.C.); eleonora.cecchin@cro.it (E.C.); riccardo.cecchin@cro.it (R.C.); ececchin@cro.it (E.C.); 2 Doctoral School in Molecular Medicine, Department of Medicine, University of Udine, 33100 Udine, Italy; 3 Clinical Trial Office, Centro di Riferimento Oncologico di Aviano (CRO), IRCCS, 33081 Aviano, Italy; sara.speziani@cro.it; 4 Division of Medical Oncology and Immune-Related Tumors, Centro di Riferimento Oncologico di Aviano (CRO), IRCCS, 33081 Aviano, Italy; arianna.dri@cro.it (A.D.); giorgia.bortolus@cro.it (G.B.); mspina@cro.it (M.S.); ssantarossa@cro.it (S.S.); lfratino@cro.it (L.F.); 5 Department of Medicine, University of Udine, 33100 Udine, Italy; fabio.puglisi@cro.it; 6 Department of Medical Oncology, Centro di Riferimento Oncologico di Aviano (CRO), IRCCS, 33081 Aviano, Italy

**Keywords:** mass spectrometry, analytical validation, therapeutic drug monitoring, prostate cancer, ARPIs

## Abstract

Accurate measurement of androgen receptor pathway inhibitors (ARPIs) and their active metabolites is essential for pharmacokinetic studies and therapeutic drug monitoring (TDM) in patients with prostate cancer (PC). However, their simultaneous determination in human plasma is analytically challenging because of the wide concentration ranges. This study aimed to develop and validate a sensitive and robust LC–MS/MS method for the quantification of abiraterone, Δ4-abiraterone, enzalutamide, N-desmethyl enzalutamide, darolutamide, keto-darolutamide, apalutamide, and N-desmethyl apalutamide in human plasma. Sample preparation was performed by protein precipitation, followed by chromatographic separation and detection using multiple reaction monitoring with isotopically labeled internal standards (total run time 6.5 min). The method was validated in accordance with regulatory guidelines by assessing selectivity, linearity, sensitivity, accuracy, precision, recovery, matrix effects, and stability. The assay demonstrated good linearity (≥0.997) across clinically relevant concentration ranges, with lower limits of quantification between 0.1 and 40 ng/mL, depending on the analyte. Intra- and inter-day precision and accuracy were within acceptable limits, and recovery and matrix effects were consistent across different plasma matrices. Stability experiments conducted in plasma and whole blood provided practical guidance for sample handling. The method was successfully applied to 79 plasma samples from 61 patients with metastatic PC. Measured concentrations were generally consistent with published pharmacokinetic data, while unexpectedly high ABI levels were observed. Sample collection occurred between 1 and 28 h after the last drug intake, enabling assessment of the analytical method across the entire pharmacokinetic profile.

## 1. Introduction

**Figure 1:**
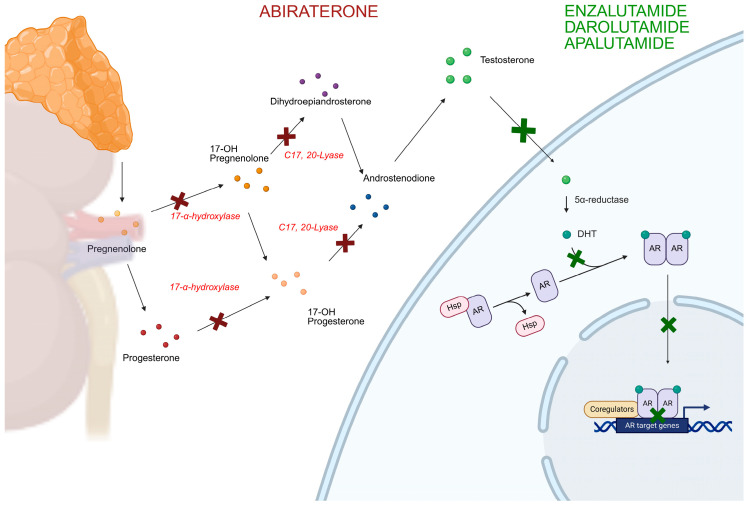
Schematic representation of the mechanisms of action of abiraterone, apalutamide, enzalutamide, and darolutamide. Red crosses denote the sites of action of abiraterone, whereas green crosses indicate the targets of the other androgen receptor pathway inhibitors (ARPIs). AR: androgen receptor; Shp: Heat Shock Protein; DHT: dihydrotestosterone.

Prostate cancer (PC) is one of the most commonly diagnosed malignancies and a leading cause of cancer-related death among men worldwide, particularly in Western countries [1]. Progression is tightly linked to androgen receptor (AR) signaling, which continues to drive tumor growth even under conditions of castrate testosterone levels. Androgen deprivation therapy (ADT) is the backbone of systemic treatment for PC. The introduction of chemotherapy and androgen receptor pathway inhibitors (ARPIs) in association with ADT has considerably improved outcomes in both metastatic hormone-sensitive PC (mHSPC) and metastatic castration-resistant PC (mCRPC) [2,3]. ARPIs are a class of oral anticancer agents targeting the AR signaling axis, which includes the androgen-biosynthesis inhibitor abiraterone acetate (ABI) and direct AR antagonists such as apalutamide (APA), darolutamide (DARO), and enzalutamide (ENZA) [3] (Figure 1).

These drugs are administered at fixed doses; however, their pharmacokinetics exhibit substantial inter-individual variability due to differences in absorption, hepatic metabolism, comorbid conditions, and frequent polypharmacy in this often elderly population [4,5,6,7,8,9,10,11]. Among the different ARPIs, the relationship between exposure and efficacy or toxicity has been extensively investigated, especially for ABI. A threshold of drug exposure (expressed as the minimum plasma concentration at the steady state (C_min_) of 8.4 ng/mL was identified as predictive of prostate-specific antigen (PSA) response [12]. Generally, no association between exposure and incidence of adverse events (AEs) has been demonstrated for ABI [12,13,14], even if one study, conducted on Japanese patients, proposed a C_min_ cutoff of a 20.6 ng/mL plasma concentration for predicting toxicity [15].

As such, therapeutic drug monitoring (TDM) has been proposed as a promising tool to optimize ABI dosing, where maintaining optimal plasma concentrations may reduce the risk of treatment failure or toxicity [16,17]. In addition, the feasibility of TDM in clinical practice has been demonstrated for ABI with the application of a cost-neutral approach to improve plasma exposure based on its food effects [13]. Conversely, further studies are needed to deepen the exposure–response relationship for ENZA, APA, and DARO [9,18,19]. However, TDM may still play an important role for these drugs in monitoring patients’ adherence to therapy and drug–drug interactions.

Despite increasing interest in applying TDM to improve the safety and effectiveness of ARPIs, analytical challenges remain. Most published methods focus on quantifying one or two of these agents, often without including their active metabolites, which can significantly contribute to overall pharmacologic activity [20,21,22,23,24,25,26,27]. A comparative table summarizing published analytical methods is reported in Supporting Information (Table S1). To our knowledge, no validated LC–MS/MS method currently allows the simultaneous quantification of ABI, ENZA, APA, DARO, and all their clinically relevant metabolites in human plasma. This analytical gap limits the feasibility of comprehensive pharmacokinetic assessment in real-world settings and clinical studies.

The aim of our work is to develop and validate, according to FDA and EMA guidelines [28,29], a method to perform TDM of ABI, ENZA, DARO, APA and their active metabolites in patients’ plasma samples. In particular, the active metabolites abiraterone Δ4 (D4A), N-desmethyl enzalutamide (N-desmethyl ENZA), keto-darolutamide (keto-DARO) and N-desmethyl apalutamide (N-desmethyl APA) show similar potency and reach similar plasma concentrations compared to the parent compound (with the exclusion of D4A) [7,14,30,31]. This method will allow for the performance of TDM of ARPIs, considering the concentrations (ideally as C_min_) of both the analytes and their active metabolites. Among the papers reported in Table S1, none simultaneously analyzed all four analytes with their active metabolites.

## 2. Results and Discussion

### 2.1. LC-MS/MS Method

**Figure 2:**
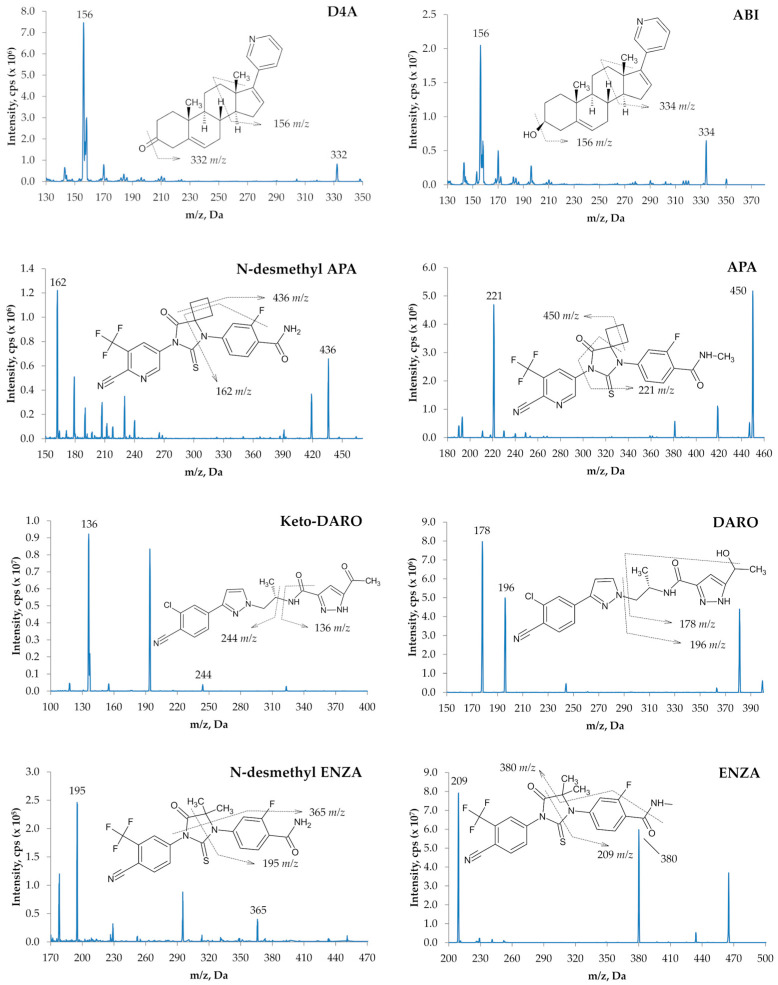
Molecular structures and proposed fragmentation of ABI, D4A, APA, N-desmethyl-APA, DARO, keto-DARO, ENZA, and N-desmethyl-ENZA.

The optimized compound-dependent parameters (especially collision energy (CE) and declustering potential (DP)) required detuning for all analytes except ABI, D4A and keto-DARO, due to detector saturation in the spectrometer. Reducing the analyte concentration during sample preparation or the final dilution step was not feasible, as this would have compromised sensitivity for ABI and D4A. The optimized and detuned values implemented in the MS method are summarized in Table S2. Quantification was conducted using the following transitions: 350 > 165 *m*/*z* for ABI, 348 > 156 *m*/*z* for D4A, 478 > 450 *m*/*z* for APA, 464 > 436 *m*/*z* for N-desmethyl-APA, 399 > 178 *m*/*z* for DARO, 397 > 244 *m*/*z* for keto-DARO, 465 > 209 *m*/*z* for ENZA, 451 > 195 *m*/*z* for N-desmethyl-ENZA. A second transition for each analyte was also chosen as a qualifier ion (Table S2). The mass fragmentation pattern for each analyte is reported in Figure 2, proposed on the basis of previously published methods [20,26,32,33,34].

The source temperature was set at 550 °C, and the ion spray voltage was 5500 V. Nitrogen was employed as nebulizer gas (50 psi), heater gas (50 psi), curtain gas (CUR) (35 psi) and collision gas (CAD) (at medium intensity).

**Figure 3:**
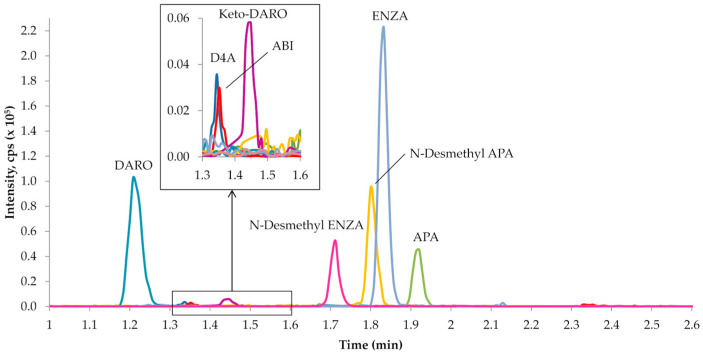
Representative chromatogram of LLOQ sample. Chromatogram shows the signal for DARO (1.22 min), D4A (1.32 min), ABI (1.35 min), keto-DARO (1.45 min), N-desmethyl-ENZA (1.73 min), N-desmethyl-APA (1.82 min), ENZA (1.84 min), and APA (1.92 min).

The chromatographic conditions allowed for an adequate peak separation, with the approximate retention times of each analyte as follows: ABI—1.35 min, D4A—1.32 min, APA—1.92 min, N-desmethyl-APA—1.82 min, DARO—1.22 min, keto-DARO—1.45 min, ENZA—1.84 min and N-desmethyl-ENZA—1.73 min (Figure 3).

### 2.2. Validation Procedure

#### 2.2.1. Recovery and Matrix Effect

Protein precipitation achieved high extraction efficiency for all analytes from the plasma matrix, with recovery ranging from 94% to 103%. For APA, ENZA, DARO, and their metabolites, the results were reproducible across both low and high concentrations (QCL and H), with SD values consistently ≤10 ng/mL and CV% ≤ 9%. Greater variability was observed for ABI and Δ4ABI, although still within the acceptance criteria, with SD values ≤ 14 ng/mL and CV% ≤ 13.8%. The recovery percentages are summarized in Table S3.

As part of the matrix effect evaluation, compound quantification using matrices from 7 healthy donors demonstrated consistent precision and accuracy, with CV% values ≤ 14.0% and accuracy ranging from 86% to 112%. When excluding ABI and D4A, the CV% was further reduced to ≤9.3%, and accuracy ranged between 91% and 112%. In addition, quantification was not influenced by hemolyzed matrices, showing accuracy between 85% and 109% with CV% ≤ 9.3%. Detailed results on accuracy and precision for each analyte across all matrices are provided in Table S4.

#### 2.2.2. Selectivity and Linearity

The analysis of each blank plasma sample from 6 healthy donors showed the absence of interfering components, demonstrating good selectivity of the method.

The good linearity was evidenced by the correlation coefficients obtained for each analyte, which were always ≥0.997. This result was also confirmed by the data on the accuracy and precision of the calibration curves: accuracy ranged from 91 to 107% for all compounds, while precision was ≤7.8%, excluding LLOQ of ABI, which has a mean CV of 19% (Table S5).

#### 2.2.3. Intraday- and Interday Precision and Accuracy

Accuracy and precision were within the acceptance criteria for both intraday and interday tests. For all compounds, intraday accuracy ranged from 85% (84% for ABI at LLOQ level) to 110%, while precision was ≤12.9% (16.1 for ABI at LLOQ level) (Table S6). In Table 1, data of interday data are reported and, considering all the analytes, interday accuracy ranged from 90 to 106%, while precision was ≤11.6%.

Reinjection reproducibility for all the analytes was demonstrated by accuracy values ranging from 86% to 103%, and precision was always lower than 10.2% (Table S7).

#### 2.2.4. Carryover

Carryover was evaluated during the accuracy and precision analysis. Initially, a non-negligible carryover was observed for ABI and DARO using a needle wash solution consisting of 70% MeCN, 30% H_2_O, and 1% formic acid. The needle wash was subsequently replaced with a solution consisting of 80% MeCN, 20% H_2_O, and 0.05% NH_3_, which improved residue removal and effectively reduced carryover. The area of the carryover in the blank sample after the ULOQ compared with the analyte response at the LLOQ was 1.1% for ABI, 7.3% for D4A, 6.6% for APA, 5.4% for N-desmethyl APA, 1.6% for DARO, 6.2% for keto-DARO, 4.1% for ENZA, and 8.0% for N-desmethyl ENZA. The area of the interfering components compared with the response of the ISs was less than 0.3%.

#### 2.2.5. Stability and Reinjection Reproducibility

Table 2 summarizes the stability of the analytes under different storage and handling conditions across various matrices. In human plasma, all analytes remained stable after 5 freeze–thaw cycles at −80 °C. Following sample processing, all compounds were also stable under autosampler conditions (4 °C) for up to 5 days.

Bench-top stability was evaluated in both plasma and whole blood. In plasma, all analytes were stable for 4 h at room temperature (RT), except for APA, which showed stability for only 2 h at RT. Based on the accuracy values observed for ABI, D4A, APA, and N-desmethyl APA (87–92%), plasma samples should be maintained at RT for a limited period to avoid underestimation of analyte concentrations.

**Figure 4:**
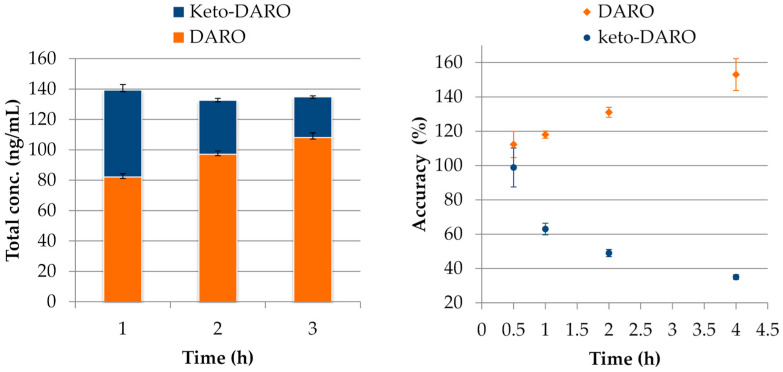
Right panel: accuracy values of DARO and keto-DARO at the QCL concentration level in whole blood after 0.5, 1, 2 and 4 h at room temperature (with respect to concentrations measured at T0); left panel: total concentration of DARO and keto-DARO at the QCL concentration level in whole blood at T0 and after 1 and 2 h at room temperature. N = 3 at each stability time.

The stability of analytes in whole blood was tested under RT or at 4 °C after 0.5, 1, 2, and 4 h. ENZA, N-desmethyl-ENZA, APA and N-desmethyl-APA were both stable at RT and at 4 °C for 4 h (acc% range 91–105%, CV% ≤ 10.4%); ABI and D4A were stable for 2 h under RT and 4 °C (acc% range 100–109%, CV% ≤ 7.9%). A separate discussion is warranted for DARO and its metabolite, keto-DARO. At low concentrations, we observed a rapid increase in DARO levels, up to 153% of the initial value after 4 h at RT (Table S8). Under the same conditions, keto-DARO concentrations decreased, showing only 35% of the initial value after 4 h at RT. A similar trend was noted during storage at 4 °C, although the effect was considerably less pronounced. This behavior appears to result from the back-conversion of keto-DARO to DARO, as previously reported at low concentrations [35]. This hypothesis is further supported by the observation that the sum of DARO and keto-DARO concentrations remained essentially constant over time (Figure 4). Thus, DARO and keto-DARO were considered stable in whole blood for only 30 min at both RT and 4 °C. In contrast, at higher concentrations, both DARO and keto-DARO remained stable after 4 h at both RT and 4 °C.

As related to long-term stability, the analytes were stable in plasma stored at −80 °C for up to 15 days (Table 2).

#### 2.2.6. Dilution Integrity

The dilution integrity for ABI and D4A, using dilution factors of 2 and 5, was successfully demonstrated, with accuracy ranging from 98% to 104% and precision consistently below 4.2% (Table 2).

#### 2.2.7. Application of the Method to Clinical Samples

Overall, 61 patients with metastatic prostate cancer were enrolled in the study. Of these, 21 (34%) received apalutamide, 17 (28%) received enzalutamide, 13 (21%) received abiraterone, and 10 (16%) received darolutamide. ARPIs were administered in the metastatic hormone-sensitive prostate cancer (mHSPC) setting in 35 patients (57%) and in the metastatic castration-resistant prostate cancer (mCRPC) setting in 26 patients (43%). Concomitant androgen deprivation therapy (ADT) consisted of leuprorelin in 29 patients (69%), triptorelin in 16 (26%), degarelix in 2 (3%), and relugolix in 1 patient. The number of patients and biological samples collected for each ARPI is reported in Table 3. Drugs were administered at approved daily doses according to prescribing information: ABI at 1000 or 500 mg; APA at 60, 120, 180, or 240 mg; DARO at 600 or 1200 mg (administered as 300 or 600 mg twice daily); and ENZA at 80, 120, or 160 mg.

**Figure 5:**
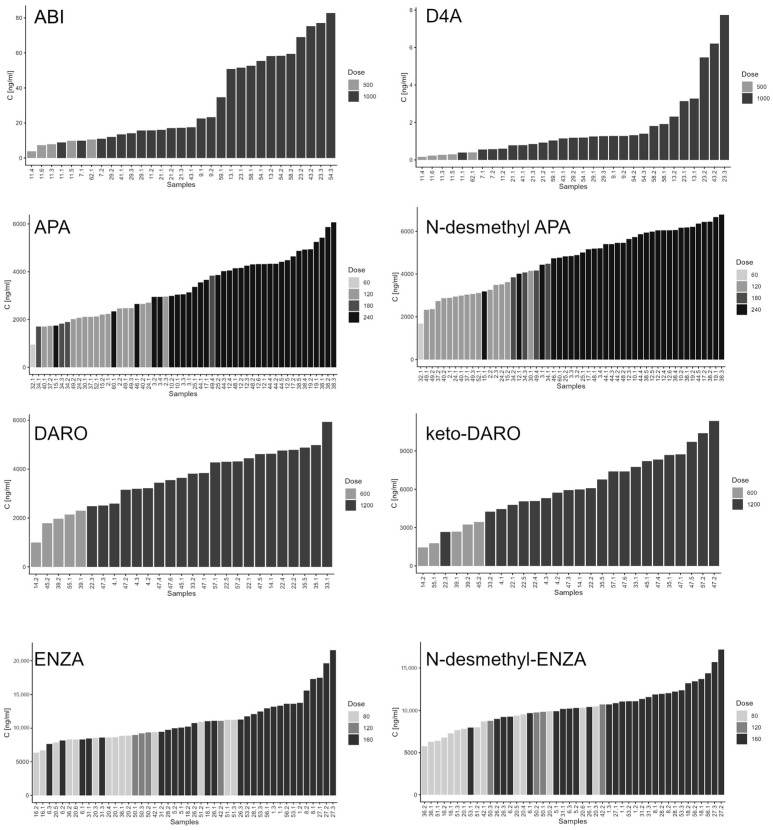
Bar plots showing ABI, D4A, APA, N-desmethyl APA, DARO, keto-DARO, ENZA, and N-desmethyl ENZA concentration for each sample, ordered from the lowest to the highest value; samples are color-coded according to the administered dose at the time of collection. Samples were labeled by the patient’s sequential identifier number and a second number denoting the specific blood draw, enabling unique linkage of each value to both the subject and the specific sampling date.

**Figure 6:**
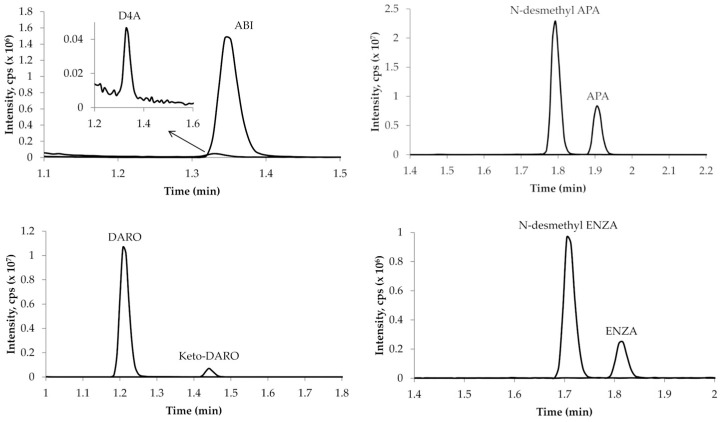
Representative chromatograms of samples collected from patients treated with ARPIs. The corresponding concentrations were as follows: 55.3 ng/mL of ABI and 1.2 ng/mL of D4A; 2818 ng/mL of APA and 6026 ng/mL of N-desmethyl APA; 2591 ng/mL of DARO and 3141 ng/mL of keto-DARO; 1725 ng/mL of ENZA and 1820 ng/mL of N-Desmethyl ENZA.

Sample collection did not always occur precisely at the expected C_min_ time point (Table S9). The median time from the last drug intake to sampling was 21 h (range: 3–28 h) for ABI, 19.5 h (range: 1–26.5 h) for ENZA, 12 h (range: 3–25 h) for DARO, and 23 h (range: 1–28 h) for APA. This variability enabled assessment of the analytical method across the entire pharmacokinetic profile, rather than being limited to C_min_. Measured plasma concentrations of each drug and their corresponding metabolites are shown in Figure 5, while examples of chromatograms are reported in Figure 6.

For D4A, ENZA, APA, DARO, and their respective metabolites, all measured concentrations fell within the validated linearity ranges of the assay. In contrast, ABI showed a different profile: measured concentrations ranged from 3.84 to 82.80 ng/mL, and 11 of 31 samples required dilution prior to analysis. These unexpectedly higher ABI plasma concentrations compared with those reported in the literature (Table 4) cannot be attributed to differences in sampling time, as 7 of the 11 out-of-range samples were collected between 21 and 24 h after the last dose. A possible explanation is incorrect drug intake with food, which is known to increase ABI plasma exposure [36]. However, this hypothesis was excluded, as information on drug intake conditions was collected at each blood sampling and confirmed that all patients took ABI according to the prescription: on an empty stomach (at least 2 h after eating or 1 h before the next meal). This finding will be further investigated once patient enrollment is completed and additional data become available.

The mean C_min_ ± SD, calculated using only samples collected at the appropriate time for C_min_ determination (24 ± 2 h for ABI, APA, and ENZA; 12 ± 1 h for DARO), is reported in Table 4. In the absence of a well-established therapeutic window for efficacy and safety for most of the study drugs, comparisons were made against population mean C_min_ values reported in the literature. An exception is ABI, for which a C_min_ threshold of 8.4 ng/mL has been identified as predictive of PSA response [12]. In our study, only one patient receiving ABI had a C_min_ below this threshold, which is consistent with the generally high ABI plasma concentrations observed. For the other ARPIs, both drug and metabolite C_min_ values were comparable to those previously reported in the literature (Table 4).

## 3. Materials and Methods

### 3.1. Standards and Chemicals

Analytical reference standards of ABI, D4A, [D_7_]-ABI, ENZA, N-desmethyl ENZA, [D_6_]-ENZA, DARO, keto-DARO, [D_4_]-DARO, APA, N-desmethyl APA and [C_13_D_3_]-APA were purchased from Alsachim (Illkirch-Graffenstaden, France). All of them, except for the stable isotopes labeled internal standards (IS), were also purchased from Targetmol chemicals Inc. (Boston, MA, USA). Dimethyl sulfoxide (DMSO) and formic acid (FA) were purchased from Sigma-Aldrich Co. (Milan, Italy); LC-MS-grade methanol (MeOH) and LC-MS-grade acetonitrile (MeCN) were purchased from Carlo Erba (Milan, Italy); type 1 ultrapure water was obtained from a Milli-Q Advantage A10 system (Millipore, Billerica, MA, USA). Control human plasma/K_2_-EDTA, used to prepare daily calibration curve and quality control samples (QCs) for the validation study, was prepared by mixing plasma samples from 10 healthy volunteers to reduce the bias of the variability between matrices. Each blank plasma sample was provided by the transfusion unit of the National Cancer Institute (Aviano, Italy).

### 3.2. Preparation of Stock and Working Solutions

Two independent sets of stock solutions (SS) (one for the calibration curve and one for the QCs) for each standard were prepared in DMSO (ABI, D4A, ENZA and N-desmethyl ENZA) or MeOH (DARO, keto-DARO, APA and N-desmethyl APA). The stock concentrations were as follows: 0.1 mg/mL for ABI and D4A; 3.00 mg/mL for ENZA, DARO and APA; and 2.00 mg/mL for N-desmethyl ENZA, keto-DARO and N-desmethyl APA. To generate the calibrator working solutions (nine solutions, from I to A), the first set of stock solutions was mixed and diluted with MeOH. The stock solutions belonging to the second set were also mixed and diluted with MeOH, obtaining three working solutions for the preparation of QCs (L-low, M-medium, H-high) (Table S10). Stock solutions of each internal standard (ISs) were prepared at 0.1 mg/mL in DMSO. All the IS stock solutions were mixed and diluted with MeCN, obtaining the IS working solution with a final concentration of 100 ng/mL. All the solutions were stored in polypropylene tubes at −80 °C.

### 3.3. Calibrators, Quality Control and Patient Sample Preparation

Calibrators and QCs were prepared by spiking the corresponding working solutions (WS) to plasma at a ratio of 1:20. The final concentrations in plasma are reported in Table 5. Each patient sample was thawed at room temperature, vortexed for 10 s, and centrifuged at 13,000× *g* for 20 s. A volume of 100 µL of either the patient, calibrator, or QC plasma was transferred into a 1.5 mL polypropylene Eppendorf tube (Eppendorf, Hamburg, Germany), followed by the addition of 500 µL of ISs working solution (100 ng/mL). The mixture was vortexed for 20 s and subjected to protein precipitation via centrifugation at 16,200× *g* for 10 min at 4 °C. Subsequently, 200 µL of the supernatant was transferred to autosampler vials. A total of 2 µL was injected into the LC–MS system for analysis.

### 3.4. Chromatographic Conditions

The HPLC system consisted of a Nexera LC-40 X3 composed of a SIL-40C X3 CL auto-sampler, three LC-40D X3 CL pumping modules, two DGU-405 CL degassers, a CBM-40 CL system controller and a CTO-40C CL column oven (Shimadzu Corporation, Tokyo, Japan). Samples were separated on a Kinetex 2.6 μm (80 Å, 50 × 2 mm) chromatographic column (Phenomenex, Torrance, CA, USA) and thermostatically controlled at 30 °C. The flow rate was kept constant at 0.4 mL/min following a multi-step gradient elution: (1) 70% MPA (initial condition); (2) from the initial condition to 10% MPA over 2.5 min and kept constant for 1.5 min; (3) from 10% MPA to initial condition over 0.5 min; (4) reconditioning for 2 min. The mobile phases (MPs) used for chromatographic separation were 0.1% HCOOH in H_2_O (MPA) and 0.1% HCOOH in MeCN (MPB). The total run time was 6.5 min.

### 3.5. Mass Spectrometric Conditions

The HPLC system was coupled to a triple quadrupole mass spectrometer, CITRINE 6500 QTrap (SCIEX, Marlborough, MA, USA). The instrument was equipped with a Turbo Ion Spray source operating in positive ion mode, and the optimization of source-dependent parameters was performed via flow injection analysis with a 100 ng/mL solution of ABI in 0.1% HCOOH in H_2_O/MeCN (1:1 *v*/*v*). Compound-dependent parameters were instead optimized via direct infusion of a 100 ng/mL solution in 0.1% HCOOH in H_2_O/MeCN (1:1 *v*/*v*) for each analyte at a flow rate of 10 μL/min. Each quantitative analysis was performed using multiple reaction monitoring (MRM) by following two MRM transitions for each analyte: one transition was exploited for quantification (quantifier), while the other was monitored in order to increase specificity (qualifier). All data were processed using Analyst MD 1.7.3 software, while peak integration was performed using the MultiQuant MD 3.0.3. software package (AB SCIEX).

### 3.6. Validation Study

A full validation study was performed according to the EMA guidelines to validate analytical methods [29].

#### 3.6.1. Recovery and Matrix Effect

Evaluation of extraction efficiency (recovery) was conducted by comparing the mean area ratio obtained from samples produced by spiking the matrix and that obtained by spiking the extracted matrix (post-extraction). This analysis was performed at two QC levels (H-high and L-low) and prepared in triplicate. Recovery results were considered acceptable if they were consistent and reproducible (CV% should not exceed 15%).

Matrix effects may arise from various sample components, including, but not limited to, endogenous biological substances such as phospholipids, carbohydrates, endogenous metabolites (e.g., bilirubin), as well as an altered protein profile (e.g., increased acute-phase proteins or immunoglobulins). These interfering substances can cause ion enhancement or suppression during analysis. The matrix effect was therefore quantitatively assessed, although the use of isotopically labeled internal standards is expected to mitigate this phenomenon. Specifically, the impact of matrix effect on the accuracy and precision of the quantification method was evaluated using 3 replicates of low and high QCs, each prepared with plasma from 8 different healthy donors, including one sample with mild hemolysis. The degree of hemolysis was visually assessed via comparison with a standardized color chart. Evaluation of hemolysis was included because rupture of red blood cells may occur during the pre-analytical phase as a result of improper blood collection or sample handling, potentially affecting analyte quantification [39].

#### 3.6.2. Selectivity and Linearity

Selectivity for analytes and ISs was evaluated using blank plasma samples obtained from 6 healthy donors. The method was considered selective if responses attributable to interfering components were less than 20% of the analyte peak area at the LLOQ (less than 5% of the IS response) for each matrix.

A nine-point plasma calibration curve was freshly prepared (Section 3.3) every day during the validation study (the concentrations of calibrators and QCs are listed in Table 5). Linearity was evaluated using two replicates of the nine calibrators in 3 analytical runs (on 3 different days). A linear regression model with a weighting factor of 1/x^2^ was applied [40]. The accuracy of the back-calculated concentrations of each calibrator should be within ±15% of the nominal concentration (20% at the LLOQ). At least 75% of the calibrators should meet the above criteria.

#### 3.6.3. Intraday and Interday Precision and Accuracy, Reinjection Reproducibility

Intraday and interday precision (calculated as CV%) and accuracy were determined by analyzing 5 replicates of LLOQ, QCL, M, and H samples. Intraday precision and accuracy were assessed on a single working day, and the test was repeated 3 times (i.e., 3 analytical runs) on 3 different days; the interday precision and accuracy were determined as the mean values of the 3 runs. The measured concentrations should be within ±15% of the nominal value, with precision ≤ 15%. At the LLOQ level, the accuracy should be between 80% and 120%, with a precision of ≤20%. One run of intraday accuracy and precision assessments was reinjected, and the viability of the processed samples was evaluated through the precision and accuracy of the reinjected QCs (reinjection reproducibility).

#### 3.6.4. Carryover

Carryover was evaluated as the percentage of the peak area of blank plasma samples injected after the upper limit of quantification (ULOQ) relative to the peak area of the LLOQ for each analyte. The carryover should not exceed 20% of the LLOQ.

#### 3.6.5. Stability and Reinjection Reproducibility

Stability evaluations under storage and handling conditions were carried out using QCL and QCH prepared in triplicate. Stability was verified if the mean concentration at each QC level was within ±15% of the nominal concentration. Freeze (−80 °C)–thaw stability was evaluated after multiple cycles. Particular attention was given to bench-top stability in both plasma and whole blood, considering the previously reported low stability of ABI in patient samples at room temperature (RT) and at 2–8 °C [21,24]. Plasma QCs were prepared as described in Section 3.3, kept at room temperature (RT) for 2 and 4 h, and subsequently analyzed. Whole-blood stability was assessed by spiking whole blood with WSs (QCL and QCH) at a blood-to-WS ratio of 20:1 (*v*:*v*). The mixture was incubated for 30 min at 37 °C and then divided into nine aliquots (300 µL each). One aliquot was immediately centrifuged (3600× *g*, 10 min, 4 °C) to obtain plasma, which was processed as described in Section 3.3 and analyzed (T0). Four aliquots were kept at RT for 0.5, 1, 2, and 4 h, while the remaining four were stored at 4 °C for the same time intervals. After incubation, all aliquots were centrifuged, and the resulting plasma was processed and analyzed as described above. For whole-blood samples only, stability was verified if the mean concentration at each QC level was within ±15% of the concentration measured at T0. Autosampler stability (AS, 4 °C) was conducted after 5 days. Long-term stability in plasma was assessed by storing the QC samples at −80 °C for 15 days.

#### 3.6.6. Dilution Integrity

The concentration range of the calibration curve for each analyte was established to encompass all expected plasma concentrations in patient samples, from C_max_ to C_min_. Initially, the assessment of dilution integrity was considered unnecessary, with the option to evaluate it later once the first set of patient samples had been analyzed. As anticipated, all patient samples showed analyte concentrations within the established calibration ranges, with the exception of ABI. The measured plasma concentrations of both compounds were significantly higher than expected, in some cases exceeding the ULOQ. Consequently, dilution integrity was assessed for ABI and D4A using a WS at a concentration of 1600 ng/mL for both compounds. The concentrations in plasma were 80 ng/mL (ULOQ 50 ng/mL for ABI and D4A) for both analytes, and the applied dilution factors were 2 and 5. The dilutions were performed using pooled blank plasma. Five replicates per dilution factor were analyzed in a single run, and both precision (≤15%) and accuracy (85–115%) were evaluated.

#### 3.6.7. Application of the Method to Clinical Samples

The proposed method was applied to quantify abiraterone, enzalutamide, darolutamide, and apalutamide (and their metabolites) in patients included in a clinical trial (protocol ID: CRO-2025-12, approval date: 28 October 2025; Parere CEUR-2025-Os-66, NCT07344363) conducted at the IRCCS National Cancer Institute CRO Aviano (Italy). The study adhered to the principles outlined in the Declaration of Helsinki, with all participants providing written informed consent. Data on the time of last drug intake and treatment adherence were recorded for all study participants. When possible, sampling was performed at C_min_. The half-life values (t_1/2_) and time (in days) to reach the steady state are reported for each drug in Table S9. The C_min_ was expected to occur approximately 24 h after the last dose for ABI, ENZA, and APA and 12 h after the last dose for DARO. Blood samples were immediately centrifuged at 3800× *g* for 10 min at 4 °C to obtain plasma. The resulting plasma was stored at −80 °C.

## 4. Conclusions

In this study, we developed and fully validated a robust LC–MS/MS method for the simultaneous quantification of ABI, ENZA, APA, DARO, and all their clinically relevant active metabolites in human plasma in accordance with current EMA and FDA bioanalytical guidelines. To our knowledge, this is the first analytical method enabling the comprehensive assessment of all four ARPIs together with their active metabolites within a single run, addressing an unmet analytical need in the context of TDM in prostate cancer.

This method demonstrated adequate selectivity, linearity, accuracy, and precision across a wide concentration range, covering both trough and peak plasma levels observed in clinical practice. Recovery was nearly complete for all analytes, which was particularly advantageous for the quantification of ABI and D4A at the very low LLOQ (0.1 ng/mL). Matrix effects were ruled out using plasma from seven healthy donors, including one hemolyzed sample. The impact of hemolysis on quantification was specifically evaluated, as this condition may occur during the pre-analytical phase due to improper blood collection or sample handling.

Particular attention was given to pre-analytical and stability aspects, including whole-blood handling, revealing drug-specific behaviors such as the back-conversion of keto-DARO to DARO at low concentrations. Specifically, samples collected from patients treated with DARO should be kept on ice and processed (centrifuged to obtain plasma) within 30 min. However, since the measured concentrations of both DARO and keto-DARO in patient samples were consistently above 1000 ng/mL, and back-conversion was observed only at low concentrations (approximately 50 ng/mL), strict adherence to these conditions may not be essential in routine practice, although they should still be taken into consideration.

Application of the method to plasma samples from patients with mCRPC and mHSPC confirmed its suitability for real-world clinical use and allowed characterization of plasma exposure to ARPIs and their metabolites across different dosing regimens. Measured concentrations were generally consistent with published pharmacokinetic data, while unexpectedly high ABI levels observed in a subset of patients warrant further investigation and highlight the potential value of TDM in identifying atypical exposure profiles. Variability in plasma concentrations among samples and patients was markedly high for ABI and its metabolite (>70% for both), whereas it was lower for APA, DARO, and ENZA (approximately 25–40%). Nevertheless, this degree of variability remains clinically relevant and underscores the need to further elucidate the exposure–response relationships of these drugs.

Overall, this method represents a valuable tool for both clinical and research applications, enabling comprehensive pharmacokinetic evaluations and supporting future studies aimed at clarifying exposure–response relationships, optimizing dosing strategies, and improving individualized treatment of prostate cancer patients receiving ARPIs.

## Figures and Tables

**Table 1:** Interday precision (CV%) and accuracy (Acc%) of ABI, D4A, ENZA, N-desmethyl ENZA, DARO, keto-DARO, APA, and N-desmethyl APA. N = 15 at each concentration level.

Analyte	Nominal Conc. (ng/mL)	Mean ± SD (ng/mL)	Acc%	CV%
ABI	0.10	0.09 ± 0.01	90	11.6
	0.25	0.24 ± 0.02	95	6.8
	18.75	17.94 ± 0.88	96	4.9
	37.50	35.98 ± 1.46	96	4.1
D4A	0.10	0.11 ± 0.01	106	9.4
	0.25	0.23 ± 0.02	94	9.6
	18.75	16.90 ± 0.63	90	3.7
	37.50	34.07 ± 1.06	91	3.1
APA	20	20 ± 1	101	5.4
	50	49 ± 3	98	5.4
	3750	3726 ± 162	99	4.4
	7500	7362 ± 235	98	3.2
N-desmethyl APA	20	19 ± 1	94	6.1
	50	53 ± 3	105	4.8
	3750	3985 ± 177	106	4.5
	7500	7446 ± 288	99	3.9
DARO	20	20 ± 1	101	3.5
	50	51 ± 3	102	5.1
	3750	3878 ± 152	103	3.9
	7500	7444 ± 146	99	2.0
keto-DARO	20	20 ± 2	101	10.3
	58	58 ± 3	100	5.6
	4300	4229 ± 185	98	4.4
	8600	8189 ± 172	95	2.1
ENZA	40	40 ± 2	99	5.3
	100	98 ± 5	97	4.9
	7500	7513 ± 313	100	4.2
	15,000	14,041 ± 308	94	2.2
N-desmethyl ENZA	40	40 ± 2	101	4.7
	100	100 ± 7	99	6.9
	7500	7486 ± 370	100	4.9
	15,000	14,745 ± 427	98	2.9

**Table 2:** Stability of the analytes under different storage/handling conditions and matrices. Only for tests in whole blood, accuracy was calculated using the mean concentration obtained at T0 as “nominal concentration”. N = 3 at each stability condition and at each concentration level (N = 5 for dilution integrity evaluation).

Analyte	Stability Condition		QC Low			QC High	
			acc%	CV%		acc%	CV%
ABI	5 freeze/thaw cycles		97	1.4		93	2
	processed sample (T = 4 °C, 5 days)		93	8.5		101	1.5
	RT in plasma (4 h)		87	6.2		87	4.4
	RT in blood (2 h)		106	7.0		100	0.9
	4 °C in blood (2 h)		106	2.8		100	4.2
	long-term stability in plasma (15 days, −80 °C)		88	9.4		97	2.3
	dilution integrity	factor 2	100	4.2	factor 5	98	0.9
D4A	5 freeze/thaw cycles		99	8.2		92	0.7
	processed sample (T = 4 °C, 5 days)		99	7.3		102	1.6
	RT in plasma (4 h)		90	3.7		88	4.4
	RT in blood (2 h)		106	7.9		106	2.6
	4 °C in blood (2 h)		109	7.6		101	3.3
	long-term stability in plasma (15 days, −80 °C)		85	10.8		97	2.3
	dilution integrity	factor 2	104	2.4	factor 5	102	2.2
APA	5 freeze/thaw cycles		91	3.7		97	2.8
	processed sample (T = 4 °C, 5 days)		108	3.4		110	2.2
	RT in plasma (2 h)		87	0.5		90	1.7
	RT in blood (4 h)		96	10.4		104	2.9
	4 °C in blood (4 h)		91	8.1		98	1.8
	long-term stability in plasma (15 days, −80 °C)		103	3.4		91	3.2
N-desmethyl APA	5 freeze/thaw cycles		103	3.2		97	3.1
	processed sample (T = 4 °C, 5 days)		113	1.4		104	1.2
	RT in plasma (4 h)		92	6.4		87	2.4
	RT in blood (4 h)		97	9.8		101	3.9
	4 °C in blood (4 h)		95	10.3		100	1.5
	long-term stability in plasma (15 days, −80 °C)		90	0.3		86	1.8
DARO	5 freeze/thaw cycles		107	1.3		104	3.5
	processed sample (T = 4 °C, 5 days)		103	4		99	1.1
	RT in plasma (4 h)		107	0.8		97	4.7
	RT in blood (30 min)		112	7.7		100	1.2
	4 °C in blood (30 min)		113	8.5		101	1.2
	long-term stability in plasma (15 days, −80 °C)		102	3.2		99	4.1
keto-DARO	5 freeze/thaw cycles		104	2.1		99	5.0
	processed sample (T = 4 °C, 5 days)		108	8		99	1.6
	RT in plasma (4 h)		109	8.3		94	4.5
	RT in blood (30 min)		99	11.3		99	2.0
	4 °C in blood (30 min)		101	11.1		100	1.1
	long-term stability in plasma (15 days, −80 °C)		108	4.1		102	5.0
ENZA	5 freeze/thaw cycles		100	0.9		98	3.8
	processed sample (T = 4 °C, 5 days)		104	3.2		104	1.8
	RT in plasma (4 h)		101	2.8		92	3.3
	RT in blood (4 h)		97	12.3		101	1.4
	4 °C in blood (4 h)		96	9.9		102	2.4
	long-term stability in plasma (15 days, −80 °C)		97	8.0		93	3.5
N-desmethyl ENZA	5 freeze/thaw cycles		100	2.7		107	2.1
	processed sample (T = 4 °C, 5 days)		105	3.1		105	1.8
	RT in plasma (4 h)		104	1.6		99	3.6
	RT in blood (4 h)		100	8.0		105	2.5
	4 °C in blood (4 h)		99	6.4		102	4.8
	long-term stability in plasma (15 days, −80 °C)		97	4.7		101	5.2

**Table 3:** Number of enrolled patients and samples collected and analyzed for each androgen receptor pathway inhibitor.

Drug	Age (Range)	Patients	Samples (N)		Posology
			Total	By Posology	
ABI	73 (59–86)	13	31	4	500 mg/die
				26	1000 mg/die
APA	73 (60–81)	21	53	15	120 mg/die
				4	180 mg/die
				33	240 mg/die
DARO	65 (53–76)	10	27	5	600 mg/die
				22	1200 mg/die
ENZA	77 (63–84)	17	46	14	80 mg/die
				4	120 mg/die
				28	160 mg/die

**Table 4:** Mean measured minimum concentration (C_min_) with corresponding standard deviation (SD) of each drug and active metabolite, with the population mean C_min_ values for study drugs and active metabolites derived from literature.

Analyte	Measured Mean C_min_ (ng/mL) ± DS	Mean C_min_ (ng/mL) from Literature	Reference
ABI	16.5 ± 13.1	11.1	[4,37]
D4A	0.83 ± 0.85	1.6	
DARO	3312 ± 1079	3780	[8,9,38]
keto-DARO	5986 ± 2843	6110	
APA	3545 ± 1217	3700	[6,7]
N-desmethyl APA	4783 ± 1354	4700	
ENZA	10,060 ± 2775	11,400	[10,11,18]
N-desmethyl ENZA	10,258 ± 1664	13,000	

**Table 5:** Analyte concentrations (ng/mL) of the calibrators and QCs.

CALIBRATORS/QCs	ABI	D4A	APA	N-Desmethyl APA	DARO	Keto-DARO	ENZA	N-Desmethyl ENZA
I	0.10	0.10	20	20	20	20	40	40
H	1.00	1.00	200	200	200	200	400	400
G	2.50	2.50	500	500	500	500	1000	1000
F	10.00	10.00	2000	2000	2000	2000	4000	4000
E	17.50	17.50	3500	3500	3500	3500	7000	7000
D	25.00	25.00	5000	5000	5000	5000	10,000	10,000
C	32.50	32.50	6500	6500	6500	6500	13,000	13,000
B	42.50	42.50	8500	8500	8500	8500	17,000	17,000
A	50.00	50.00	10,000	10,000	10,000	10,000	20,000	20,000
QCL	0.25	0.25	50	50	50	58	100	100
QCM	18.75	18.75	3750	3750	3750	4300	7500	7500
QCH	37.50	37.50	7500	7500	7500	8600	15,000	15,000

## Data Availability

The original contributions presented in this study are included in the article/Supplementary Materials. Further inquiries can be directed to the corresponding author.

## Supplementary Materials

The following supporting information can be downloaded at: https://www.mdpi.com/article/10.3390/ijms27073017/s1.
