# Supplementary material for: LC-MS/MS Method for Therapeutic Drug Monitoring of Abiraterone, Darolutamide, Apalutamide, Enzalutamide, and Metabolites in Prostate Cancer Patients
Source: Int J Mol Sci. 2026 Mar 26;27(7):3017. doi: 10.3390/ijms27073017 (PMC13073828; doi:10.3390/ijms27073017)
Supplement: Supplementary file 1 [file ijms-27-03017-s001.zip › ijms-4211042-supplementary.pdf]

Supplementary Materials

Table S1. Published LC-MS methods for the quantification of ABI, ENZA, APA, DARO, and all their clinically relevant metabolites in human plasma.

Ref	Analyte(s)	Plasma vol (μL)	Sample prep	LLOQ(ng/mL)								ULOQ(ng/mL)							
				ABI	D4A	ENZA	N-DESM ENZA	DARO	KETO-DARO	APA	N-DESM APA	ABI	D4A	ENZA	N-DESM ENZA	DARO	KETO DARO	APA	N-DESM APA
[1]	ENZA, N-desmethyl ENZA, (carboxylic acid metabolite)	50	LLE	/	/	20	20	/	/	/	/	/	/	50000	50000	/	/	/	/
[2]	ABI	200	PP	1	/	/	/	/	/	/	/	500	/	/	/	/	/	/	/
[3]	ABI, ENZA, N-desmethyl ENZA (bicalutamide)	50	PP	1	/	100	100	/	/	/	/	1000	/	30000	30000	/	/	/	/
[4]	ABI; ENZA; N-desmethyl ENZA	50	PP	1	/	5	10	/	/	/	/	100	/	500	1000	/	/	/	/
[5]	ABI; D4A; ENZA; N-desmethyl ENZA, (anastrozole, bicalutamide, Z-endoxifen, exemastane, letrozole)	50	PP	2	0.2	1500	1500	/	/	/	/	200	20	30000	30000	/	/	/	/
[6]	ABI; D4A (3-keto-5α-ABI, ABI N-oxide, ABI sulfate, ABI N-oxide sulfate)	100	PP	0.5	0.01	/	/	/	/	/	/	200	4	/	/	/	/	/	/
[7]	ABI, ENZA, DARO	25	PP	1	/	50	/	50	/	/	/	100	/	5000	/	5000	/	/	/
[8]	Apalutamide, N-desmethyl Apalutamide	50	PP	/	/	/	/	/	/	25	25	/	/	/	/	/	/	20000	20000
	ABI, D4A, ENZA, N-desmethyl ENZA, DARO, keto-DARO, APA, N-desmethyl APA	100	PP	0.1	0.1	40	40	20	20	20	20	50	50	20000	20000	10000	10000	10000	10000

PP: protein precipitation; LLE: liquid-liquid extraction; LLOQ: lower limit of quantification

Table S2. Compound-dependent parameters and ion transitions of each analyte and IS used for mass spectrometry. In brackets the original (optimized) values that were subsequently detuned for saturation issues. *Product ion used as quantifier.

Precursor Ion				Product Ion		
Analyte	Q1 (amu)	DP (volts)	EP (volts)	Q3 (amu)	CE (volts)	CXP (volts)
ABI	350.3	130	10	156.1*	74	26
				334.2	55	18
D4A	348.2	140	10	156.1*	72	25
				332.1	53	18
APA	478.1	160 (110)	10	450.0*	42 (35)	12
				221.0	35	38
N-desmethyl APA	464.2	160 (65)	10	436.2*	33	11
				161.9	55	27
DARO	399.2	115 (75)	10	178.1*	23 (31)	30
				196.2	25	33
keto-DARO	397.2	70	10	244.1*	45	20
				136.1	27	22
ENZA	465.1	40 (200)	10	209.1*	29 (39)	35
				380.1	37	9
N-desmethyl ENZA	451.1	160 (100)	10	195.2*	60 (38)	15
				365.9	36	9
[$^2H_7$]-ABI	357.4	150	10	157.1*	75	26
				341.3	56	18
[^{13}C,$^2H_3$]-APA	482.2	155	10	225.2*	35	39
				454.4	34	12
[$^2H_4$]-DARO	403.3	85	10	182.0*	31	31
				200.0	27	30
[$^2H_6$]-ENZA	471.2	160	10	215.2*	40	37
				380.1	36	9

Q1: first quadrupole mass; Q3: third quadrupole mass; DP: declustering potential; EP: entrance potential; CE: collision energy; CXP: cell exit potential.

Table S3. Percentage of recovery of ABI, D4A, ENZA, N-desmethyl-ENZA, DARO, keto-DARO, APA, and N-desmethyl-APA from human plasma. N = 3 at each concentration level.

Analyte	Nominal conc. (ng/mL)	Recovery (%) ± SD	CV%
ABI	0.25	101±13	13.2
	37.5	100±4	4.2
D4A	0.25	103±14	13.8
	37.5	99±4	4.4
APA	50.25	94±8	9.0
	7500	95±6	6.0
N-desmethyl APA	50	95±7	7.2
	7500	94±6	6.3
DARO	50	103±7	6.6
	7500	100±4	4.2
keto-DARO	58	103±9	8.4
	8600	100±5	4.6
ENZA	100	103±10	9.5
	15000	102±5	4.6
N-desmethyl ENZA	100	98±6	5.9
	15000	99±4	4.3

Table S4. Matrix effect evaluation: accuracy and precision of QCL and QCH of ABI, D4A, ENZA, N-desmethyl-ENZA, DARO, keto-DARO, APA, and N-desmethyl-APA in matrices from seven healthy donors (M1-6 and haemolysed). All the concentrations are expressed in ng/mL. N = 3 at each concentration level.

		M1			M2			M3			M4			M5			M6			Haemolysed		
Analyte	Nominal conc.	mean ±SD	Acc %	CV %	mean ±SD	Acc%	CV %	mean ±SD	Acc%	CV %	mean ±SD	Acc%	CV %	mean ±SD	Acc%	CV %	mean ±SD	Acc%	CV %	mean ±SD	Acc%	CV %
ABI	0.25	0.26±0.02	104	5.9	0.26±0.01	105	4.6	0.25±0.02	99	7.4	0.26±0.02	104	8.6	0.25±0.03	99	10.3	0.27±0.04	107	14.0	0.24±0.01	97	3.3
	37.5	37.2±1.9	99	5.1	36.9±0.1	98	0.3	37.2±1.6	99	4.3	37.3±2.1	99	5.7	36.7±1.1	98	2.9	34.6±0.9	92	2.5	34.9±0.6	93	1.8
D4A	0.25	0.27±0.01	106	5.5	0.24±0.02	98	6.4	0.24±0.03	96	11.1	0.26±0.02	104	7.5	0.24±0.01	96	4.5	0.24±0.01	96	9.7	0.23±0.01	92	3.4
	37.5	35.1±1.6	94	4.6	34.9±0.5	93	1.5	35.2±1.2	94	3.3	35.6±1.3	95	3.6	33.5±0.8	89	2.4	32.2±0.4	86	1.3	32.0±0.7	85	2.0
APA	50.25	53.6±3.1	107	5.8	51.7±3.3	103	6.4	49.3±1.4	98	2.9	51.8±2.6	103	5.0	52.0±2.1	103	4.0	50.0±0.6	100	1.2	51.0±2.6	102	5.0
	7500	7984±223	106	2.8	7910±198	105	2.5	7966±346	106	4.4	8131±418	108	5.1	7397±173	99	2.3	7313±141	98	1.9	7423±39	99	5.3
N-desmethyl APA	50.25	56.2±3.2	112	5.7	54.5±3.5	108	6.5	55.6±2.0	111	3.6	56.3±2.6	112	4.6	56.2±0.8	112	1.4	54.8±3.6	109	6.6	54.5±5.1	109	9.3
	7500	7628±168	102	2.2	7647±167	102	2.2	7898±301	105	3.8	7906±233	105	2.9	7347±129	98	1.8	7232±72	96	1.0	7387±284	98	3.8
DARO	50.25	55.7±2.5	111	4.5	54.0±2.6	107	4.7	55.5±1.3	111	2.3	54.9±2.3	109	4.1	52.2±1.2	104	2.3	53.6±2.4	107	4.6	52.5±3.8	104	7.2
	7500	7603±131	101	1.7	7615±60	102	0.8	790±392	105	5.0	7912±182	105	2.3	7495±263	100	3.5	7247±147	97	2.0	7358±131	98	1.8
keto-DARO	58	63.9±1.4	110	2.2	62.2±3.5	107	5.7	59.3±4.7	102	7.8	63.4±3.6	109	5.6	62.1±3.4	107	5.5	59.3±0.4	102	0.7	58.4±3.2	100	5.5
	8600	8481±23	99	0.3	8385±48	98	0.6	8837±344	103	3.9	8663±109	101	1.3	8196±245	95	3.0	7848±140	91	1.8	8184±78	95	0.9
ENZA	100.5	102.0±5.4	101	5.3	98.4±6.2	98	6.3	101.4±3.9	101	3.8	100.5±3.1	100	3.1	97.2±2.7	97	2.7	96.3±6.0	96	6.2	95.1±5.4	95	5.7
	15000	14540±78	97	0.5	14483±356	97	2.5	14713±225	98	1.5	14833±354	99	2.4	14080±428	94	3.0	13733±163	92	1.2	14007±140	93	1.0
N-desmethyl ENZA	100.5	103.7±5.6	103	5.4	102.8±7.6	102	7.4	97.9±1.8	97	1.8	101.6±5.4	101	5.3	96.3±6.3	96	6.6	92.9±3.2	92	3.4	95.8±0.8	95	0.9
	15000	15370±204	102	1.3	15133±305	101	2.0	15510±252	103	1.6	15650±304	104	1.9	14953±706	100	4.7	14070±171	94	1.2	14810±625	99	4.2

Table S5. Precision and accuracy of the calibration curves. N = 3 at each concentration level.

Analyte	Nominal conc. (ng/mL)	Mean ± SD	CV%	Acc%
ABI	0.1	0.10±0.02	19.0	100
	1	1.00±0.04	3.9	100
	2.5	2.50±0.10	4.0	99
	10	10.10±0.31	3.1	101
	17.5	17.70±0.52	3.0	101
	25	24.90±0.69	2.8	100
	32.5	33.00±0.85	2.6	101
	42.5	42.10±1.77	4.2	99
	50	49.40±1.62	3.3	99
D4A	0.1	0.10±0.01	6.3	100
	1	1.00±0.03	3.3	100
	2.5	2.50±0.06	2.4	99
	10	9.90±0.23	2.3	99
	17.5	17.70±0.49	2.7	101
	25	25.10±0.80	3.2	100
	32.5	33.00±0.86	2.6	101
	42.5	42.40±1.25	2.9	100
	50	49.5±1.38	2.8	99
APA	20	20.0±0.5	2.5	100
	200	196.7±7.8	4.0	98
	500	495.2±17.3	3.5	99
	2000	2043.5±98.1	4.8	102
	3500	3491.0±109.8	3.1	100
	5000	4939.3±385.3	7.8	99
	6500	6739.3±283.8	4.2	104
	8500	8373.0±202.9	2.4	99
	10000	9755.8±567.3	5.8	98
N-desmethyl APA	20	19.8±1.0	5.2	99
	200	213.6±6.4	3.0	107
	500	524.7±25.2	4.8	105
	2000	1829.5±65.9	3.1	107
	3500	3559.8±164.8	4.6	102
	5000	4967±112.8	2.3	99
	6500	6370.5±220.3	3.5	98
	8500	7862.2±326.1	4.1	92
	10000	9080.7±427.7	4.7	91
DARO	20	19.9±0.41	2.0	100
	200	204.7±3.2	1.6	102
	500	517.2±11.9	2.3	103
	2000	2064.0±40.5	2.0	103
	3500	3546.7±125.2	3.5	101
	5000	5005.8±122.5	2.4	100
	6500	6456.8±86.9	1.3	99
	8500	8115.2±272.6	3.4	95
	10000	9511.5±348.2	3.7	95
keto-DARO	20	19.9±1.3	6.5	99
	200	208.6±5.4	2.6	104
	500	520.5±17.2	3.3	104
	2000	2037.3±62.7	3.1	102
	3500	3487.8±98.2	2.8	100
	5000	4931.8±141.5	2.9	99
	6500	6476.2±145.4	2.2	100
	8500	8119.3±237.3	2.9	96

	10000	9687.8±428.8	4.4	97
ENZA	40	39.9±0.5	1.1	100
	400	408.2±11.3	2.8	102
	1000	1006.5±17.8	1.8	101
	4000	4277.5±80.8	1.9	107
	7000	7174.7±159.0	2.2	102
	10000	10147.3±338.9	3.3	101
	13000	13023.3±234.6	1.8	100
	17000	15950.0±451.5	2.8	94
	20000	18535.0±448.2	2.4	93
N-desmethyl ENZA	40	40.0±1.3	3.4	100
	400	404.6±10.2	2.5	101
	1000	974.8±17.0	1.7	97
	4000	4077.8±98.3	2.4	102
	7000	6988.7±194.1	2.8	100
	10000	10032.7±192.4	1.9	100
	13000	13161.7±248.6	2.2	101
	17000	16541.7±463.1	2.8	97
	20000	20150.0±673.1	3.3	101

Table S6. Intraday precision and accuracy data for ABI, D4A, ENZA, N-desmethyl-ENZA, DARO, keto-DARO, APA, and N-desmethyl-APA. All the concentrations are expressed in ng/mL. N = 5 at each concentration level and at each run.

		Run 1			Run 2			Run 3		
Analyte	Nominal conc.	mean ±SD	Acc %	CV %	mean ±SD	Acc %	CV %	mean ±SD	Acc %	CV%
ABI	0.1	0.09±0.01	92	16.1	0.09±0.01	95	9.2	0.08±0.00	84	2.9
	0.25	0.24±0.02	94	9.4	0.24±0.02	95	6.9	0.24±0.01	97	4.8
	18.75	17.94±0.88	101	1.0	17.03±0.29	91	1.7	17.80±0.36	95	2.0
	37.5	35.95±1.46	98	2.0	34.29±0.87	91	2.5	36.76±0.89	98	2.4
D4A	0.1	0.10±0.01	104	12.9	0.11±0.01	108	11.0	0.11±0.004	106	3.6
	0.25	0.21±0.02	85	8.4	0.25±0.01	100	3.8	0.24±0.02	89	8.2
	18.75	17.63±0.36	94	2.0	16.43±0.33	88	2.0	16.64±0.36	89	2.2
	37.5	34.80±0.87	93	2.5	32.84±0.14	88	0.4	34.55±0.55	92	1.6
APA	20	20.8±1.0	104	4.6	20.8±0.8	104	3.9	19.2±0.8	96	4.1
	50	48.2±3.0	96	6.1	50.2±2.3	100	4.6	49.3±2.8	98	5.8
	3750	3895.2±127.4	104	3.3	3666.6±105.3	98	2.9	3608.6±84.3	96	2.3
	7500	7442.6±355.4	99	4.8	7305.4±70.7	97	1.0	7337.2±221.0	98	3.0
N-desmethyl APA	20	19.3±1.3	97	6.7	19.1±0.8	95	4.3	18.1±1.1	91	6.2
	50	51.0±1.6	101	3.1	53.1±2.9	106	5.5	54.5±2.0	108	3.6
	3750	4140.0±203.3	110	4.9	3960.0±64.0	106	1.6	3853.8±111.0	108	2.9
	7500	7368.8±447.9	98	6.1	7545.8±236.4	101	3.1	7423.4±111.7	99	1.5
DARO	20	20.0±0.5	100	2.4	20.2±0.5	101	2.3	20.3±1.1	101	5.4
	50	49.9±2.1	99	4.3	49.7±1.7	99	3.3	54.0±1.2	108	2.3
	3750	4030.4±70.5	107	1.7	3726.6±98.2	99	2.6	3877.4±93.4	103	2.4
	7500	7530.0±138.4	100	1.8	7377.0±120.0	98	1.6	7423.8±160.2	99	2.2
keto-DARO	20	18.2±2.3	91	12.5	21.6±1.1	108	5.1	21.0±1.0	105	4.5
	58	57.2±3.3	99	5.8	56.2±3.7	97	6.6	59.9±1.6	103	2.7
	4300	4417.8±124.8	103	2.8	4051.0±125.9	94	3.1	4217.0±64.9	98	1.5
	8600	8293.2±228.1	96	2.8	8073.6±77.8	94	1.0	8199.2±122.6	95	1.5
ENZA	40	39.3±0.9	98	2.2	41.6±2.0	104	4.7	38.1±1.8	95	4.7
	100.	92.5±2.5	92	2.7	99.1±2.8	99	2.8	101.4±3.8	101	3.7
	7500	7835.0±123.3	104	1.6	7268.2±204.6	97	2.8	7435.4±271.1	99	3.6
	15000	14200.0±270.6	95	1.9	13858.0±258.2	92	1.9	14066.0±343.1	94	2.4
N-	40	41.0±0.7	102	1.6	39.1±1.5	98	3.8	40.9±2.7	102	6.6

desmethyl ENZA	100	94.7±4.1	94	4.3	97.1±3.2	97	3.3	107.5±5.0	107	4.6
	7500	7847.6±206.0	105	2.6	7174.4±284.1	96	4.0	7435.2±249.8	99	3.4
	15000	14798.0±540.2	99	3.7	1448.0±405.2	97	2.8	14958.0±185.0	100	1.2

Table S7. Reinjection reproducibility: accuracy and precision of reinjected QC samples. N = 5 at each concentration level.

Analyte	Nominal conc. (ng/mL)	mean ±SD (ng/mL)	Acc%	CV%
ABI	0.1	0.10±0.01	103	5.2
	0.25	0.23±0.01	91	4.2
	18.75	17.12±0.42	91	2.5
	37.5	34.65±1.11	92	3.2
Δ4A	0.1	0.10±0.01	98	7.2
	0.25	0.24±0.01	94	4.7
	18.75	16.17±0.38	86	2.4
	37.5	32.95±1.37	88	4.2
APA	20	20.4±1.3	102	6.5
	50	50.3±1.7	100	3.4
	3750	3558.6±61.7	95	1.7
	7500	7248.8±173.8	97	2.4
N-desmethyl APA	20	20.1±1.1	100	5.3
	50	55.4±2.1	110	3.7
	3750	3867.2±86.4	103	2.2
	7500	7355.8±129.6	98	1.8
DARO	20	20.0±0.3	100	1.7
	50	50.7±0.8	101	1.7
	3750	3722.6±138.5	99	3.7
	7500	7262.0±265.8	97	3.7
keto-DARO	20	18.4±1.9	92	10.2
	58	54.5±2.2	94	4.1
	4300	4032.6±129.4	94	3.2
	8600	7913.8±277.4	92	3.5
ENZA	40	40.2±0.9	100	2.3
	100	94.7±1.4	94	1.4
	7500	7139.6±176.4	95	2.5
	15000	13624.0±295.6	91	2.2
N-desmethyl ENZA	40	40.7±1.6	102	2.8
	100	94.3±3.6	94	3.8
	7500	7081.4±174.2	94	2.5
	15000	14138.0±514.8	94	3.6

Table S8. Precision and accuracy data from stability test of DARO and keto-DARO in whole blood stored at RT and 4 °C. N = 3 at each concentration level.

Stability conditions	DARO				keto-DARO			
	QC Low		QC High		QC Low		QC High	
	acc%	CV%	acc%	CV%	acc%	CV%	acc%	CV%
RT in blood (1h)	118	2.1	109	1.5	63	3.4	102	2.5
RT in blood (2h)	131	2.9	111	2.9	49	2.1	97	3.0
RT in blood (4h)	153	9.3	114	5.5	35	1.4	89	3.1
4 °C in blood (1h)	119	4.0	107	2.1	81	2.5	105	1.7
4 °C in blood (2h)	121	2.3	106	1.8	69	3.9	101	2.6
4 °C in blood (4h)	115	7.5	101	3.9	78	13.9	97	2.9

Table S9. Drug half-life ($t_{1/2}$) and timing to correctly measure the C_{min} of ABI, APA, ENZA and DARO.

drug	$t_{1/2}$	Days to reach steady state	Time from the last dose intake to reach C_{min}	Reference
ABI	12 h	8	24 h	[9,10]
ENZA	5.8 days	28	24 h	[11,12]
DARO	(S,R) 9 h<br>(S,S) 22 h	5	12 h	[13,14]
APA	3 days	28	24 h	[15,16]

Table S10. Concentrations of analytes in the working solutions, expressed in ng/mL (calibrators and QCs).

WS	ABI	Δ4A	APA	N-desmethyl APA	DARO	keto-DARO	ENZA	N-desmethyl ENZA
I	2	2	400	400	400	400	800	800
H	20	20	4000	4000	4000	4000	8000	8000
G	50	50	10000	10000	10000	10000	20000	20000
F	200	200	40000	40000	40000	40000	80000	80000
E	350	350	70000	70000	70000	70000	140000	140000
D	500	500	100000	100000	100000	100000	200000	200000
C	650	650	130000	130000	130000	130000	260000	260000
B	850	850	170000	170000	170000	170000	340000	340000
A	1000	1000	200000	200000	200000	200000	400000	400000
QCL	5	5	1005	1005	1005	1160	2010	2010
QCM	375	375	75000	75000	75000	86000	150000	150000
QCH	750	750	150000	150000	150000	172000	300000	300000

References

[1] D. Bennett, J.A. Gibbons, R. Mol, Y. Ohtsu, C. Williard, Validation of a method for quantifying enzalutamide and its major metabolites in human plasma by LC-MS/MS, Bioanalysis 6 (2014) 737–744. https://doi.org/10.4155/bio.13.325.

[2] G.E. Benoist, E. van der Meulen, F.J.E. Lubberman, W.R. Gerritsen, T.J. Smilde, J.A. Schalken, J.H. Beumer, D.M. Burger, N.P. van Erp, Analytical challenges in quantifying abiraterone with LC-MS/MS in human plasma, Biomed Chromatogr 31 (2017). https://doi.org/10.1002/bmc.3986.

[3] K.-P. Kim, R.A. Parise, J.L. Holleran, L.D. Lewis, L. Appleman, N. van Erp, M.J. Morris, J.H. Beumer, Simultaneous quantitation of abiraterone, enzalutamide, N-desmethyl enzalutamide, and bicalutamide in human plasma by LC-MS/MS, J Pharm Biomed Anal 138 (2017) 197–205. https://doi.org/10.1016/j.jpba.2017.02.018.

[4] M. van Nuland, H. Rosing, J. de Vries, H. Ovaa, J.H.M. Schellens, J.H. Beijnen, An LC-MS/MS method for quantification of the active abiraterone metabolite Δ(4)-abiraterone (D4A) in human plasma, J Chromatogr B Analyt Technol Biomed Life Sci 1068–1069 (2017) 119–124. https://doi.org/10.1016/j.jchromb.2017.10.033.

[5] M. van Nuland, N. Venekamp, N. de Vries, K. a. M. de Jong, H. Rosing, J.H. Beijnen, Development and validation of an UPLC-MS/MS method for the therapeutic drug monitoring of oral anti-hormonal drugs in oncology, J Chromatogr B Analyt Technol Biomed Life Sci 1106–1107 (2019) 26–34. https://doi.org/10.1016/j.jchromb.2019.01.001.

[6] Y. Hu, J. Wu, X. Jiang, G. Chen, Y. Zhang, L. Si, H. Jiang, J. Huang, J. Huang, Simultaneous determination of abiraterone and its five metabolites in human plasma by LC-MS/MS: Application to pharmacokinetic study in healthy Chinese subjects, J Pharm Biomed Anal 217 (2022) 114826. https://doi.org/10.1016/j.jpba.2022.114826.

[7] S.A.J. Buck, P. de Bruijn, I.M. Ghobadi-Moghaddam-Helmantel, M.H. Lam, R. de Wit, S.L.W. Koolen, R.H.J. Mathijssen, Validation of an LC-MS/MS method for simultaneous quantification of abiraterone, enzalutamide and darolutamide in human plasma, J Chromatogr B Analyt Technol Biomed Life Sci 1225 (2023) 123752. https://doi.org/10.1016/j.jchromb.2023.123752.

[8] Y. Zhu, L. Chen, W. Cheng, D. Yang, L. Zhang, J. Li, Liquid chromatography-tandem mass spectrometry assay for the simultaneous determination of apalutamide and its active metabolite N-desmethyl apalutamide, and its application in real-world patients with castration-resistant prostate cancer, Front Pharmacol 16 (2025) 1510583. https://doi.org/10.3389/fphar.2025.1510583.

[9] FDA Multi-discipline Review, Abiraterone-CLINICAL PHARMACOLOGY AND BIOPHARMACEUTICS REVIEW, (n.d.). https://www.accessdata.fda.gov/drugsatfda_docs/nda/2011/202379Orig1s000ClinPharmR.pdf (accessed February 3, 2026).

[10] K. Stuyckens, F. Saad, X.S. Xu, C.J. Ryan, M.R. Smith, T.W. Griffin, M.K. Yu, A. Vermeulen, P. Nandy, I. Poggesi, Population pharmacokinetic analysis of abiraterone in chemotherapy-naïve and docetaxel-treated patients with metastatic castration-resistant prostate cancer, Clin Pharmacokinet 53 (2014) 1149–1160. https://doi.org/10.1007/s40262-014-0178-6.

[11] EMA Product Information, Enzalutamide- SUMMARY OF PRODUCT CHARACTERISTICS, (n.d.). https://www.ema.europa.eu/en/documents/product-information/xtandi-epar-product-information_en.pdf (accessed February 3, 2026).

[12] FDA Multi-discipline Review, Enzalutamide-CLINICAL PHARMACOLOGY REVIEW, (n.d.). https://www.accessdata.fda.gov/drugsatfda_docs/nda/2020/213674Orig1s000MultidisciplineR.pdf (accessed February 3, 2026).

[13] EMA Product Information, Darolutamide- Summary of product characteristics, (n.d.). https://www.ema.europa.eu/en/documents/product-information/nubeqa-epar-product-information_en.pdf (accessed February 3, 2026).

[14] FDA Multi-discipline Review, Darolutamide-CLINICAL PHARMACOLOGY AND BIOPHARMACEUTICS REVIEW, (n.d.). https://www.accessdata.fda.gov/drugsatfda_docs/nda/2019/212099Orig1s000MultidisciplineR.pdf (accessed February 3, 2026).

[15] EMA Product Information, Apalutamide- Summary of product characteristics, (n.d.). https://www.ema.europa.eu/en/documents/product-information/erleada-epar-product-information_en.pdf (accessed February 3, 2026).

[16] FDA Multi-discipline Review, Apalutamide-CLINICAL PHARMACOLOGY REVIEW, (n.d.). https://www.accessdata.fda.gov/drugsatfda_docs/nda/2018/210951orig1s000multidiscipliner.pdf (accessed February 3, 2026).
